# Randomized controlled trial on the effects of mindfulness-based respiratory decompression therapy in claustrophobic patients undergoing MRI inspection

**DOI:** 10.3389/fmed.2023.1253824

**Published:** 2023-11-22

**Authors:** Yi Zhou, Yanqing Cao, Shan Xu, Sijin Li, Yidan Liang, Wei Zhang, Yunping Xiao

**Affiliations:** Department of Radiology, Liuzhou People's Hospital Affiliated to Guangxi Medical University, Liuzhou, China

**Keywords:** mindfulness respiratory decompression therapy, claustrophobia, MRI, self-rating anxiety scale, stress reduction function

## Abstract

**Background:**

Claustrophobia is a psychological disease. It is estimated to occur in 2.1–14.3% of all magnetic resonance imaging (MRI) examinations. Mindfulness decompression is an effective means to treat and reduce fear and anxiety. There is a rare report on the application of mindfulness-based stress reduction therapy in the magnetic resonance examinations of patients with claustrophobia to date.

**Purpose:**

The purpose of this study is to explore the intervention effect of mindfulness respiratory decompression therapy on the MRI inspection of patients with claustrophobia.

**Methods:**

A total of 86 patients with claustrophobia requiring MRI in our hospital from January 2018 to December 2020 were divided into two groups. The control group was given routine psychological nursing, and the observation group was given a mindfulness breathing technique on the basis of the control group. Before and after the intervention, we compared the intervention effect, satisfaction with nurses’ psychological intervention technique, severe autonomic nervous symptoms during the examination, self-rating anxiety scale (SAS) scores, and profile of mood states revised (POMS-R) scores.

**Results:**

The total effective rate of intervention in the observation group was 90.90%, which was significantly higher (*χ*^2^ = 6.857, *p* = 0.00004) than that in the control group (26.19%). Severe autonomic nervous symptoms in the observation group were significantly lower than those in the control group (*p* < 0.05). After the intervention, SAS scores and POMS-R scores in the observation group decreased with statistical significance (*p* < 0.05).

**Conclusion:**

Mindfulness respiratory decompression therapy can effectively help claustrophobic patients complete an MRI examination, which may be worthy of wide promotion and application in the clinic.

## 1 Introduction

Claustrophobia is a psychological disease, a type of terror neurosis, and a fear of closed space. Claustrophobia refers to severe autonomic nervous symptoms such as abnormal fear, shortness of breath, rapid heartbeat, sweating, a near-death feeling, and even syncope caused by patients entering narrow and dark spaces (1–3). Among various types of phobias, claustrophobia is more common than other phobias. This type of phobia is an unreasonable fear that occurs in enclosed spaces such as elevators, tunnels, and computed tomography and magnetic resonance imaging (MRI) equipment (3). According to a report (4), approximately 12.5% of the population has this fear, and most of them are women. It is estimated to occur in 2.1 to 14.3% of all MRI examinations (4–7). In the consensus of experts on magnetic resonance examination technology, claustrophobia is listed as a relative contraindication (8). A previous study (9) pointed out the specific diagnostic criteria of claustrophobia patients: fear of the imbalance between emotion and situation; cannot be eliminated by explanation; uncontrollable; and an avoidance response occurs when patients are in a fearful environment.

With the popularization and promotion of MRI examinations in recent years, the number of patients undergoing the examination is gradually increasing. Claustrophobia caused by MRI examinations has attracted increasing attention (10). Since 2015, the Joint Committee has also put forward new diagnostic imaging requirements for MRI safety, including claustrophobia (5). At present, the solutions for MRI examinations for claustrophobic patients at home and abroad are as follows: Sedation is used in MRI examinations, but this method has certain risks (11), and some foreign scholars have pointed out that reducing noise and increasing space in the scanner can hopefully reduce the incidence of claustrophobia (12). However, the new scanner is expensive, and most existing magnets cannot reduce noise and improve space. Desensitization technology was supplied for treating other virtual reality phobias in MRI patients (11). MRI is a costly imaging method. Optimized contact can improve nursing quality, patient satisfaction, and the magnetic resonance utilization rate (13). Psychological intervention may become a coping strategy for claustrophobia (14).

In recent years, mindfulness, as a psychological intervention method, has been increasingly applied to treat patients with mental disorders and physical diseases. Mindfulness refers to a specific, non-judgmental focus on the present situation. Mindfulness-based stress reduction was proposed by Jon Kabat-Zinn in 1979, and its main theoretical basis comes from the combination of Buddhism and modern psychology (15). It includes mindfulness breathing, mindfulness meditation, mindfulness movement, and body scanning. Through mindfulness-based stress reduction, patients can improve their mindfulness level, relieve anxiety, depression, and other negative emotions, and eventually improve their quality of life (16, 17). Many studies have shown that mindfulness decompression is an effective means to treat and reduce fear and anxiety (18–22). However, there is a rare report on the application of mindfulness-based stress reduction therapy in the magnetic resonance examinations of patients with claustrophobia to date. Therefore, to explore the effectiveness of mindfulness decompression therapy in the magnetic resonance examinations of patients with claustrophobia, researchers tried to use the therapy to provide psychological intervention to claustrophobic patients in MRI examinations.

## 2 Methods

### 2.1 Patients

From January 2018 to December 2020, 86 claustrophobic patients requiring a magnetic resonance examination were selected for a randomized controlled trial at the Liuzhou People’s Hospital Affiliated with Guangxi Medical University. Inclusion criteria are as follows: (1) meet the diagnostic criteria for claustrophobia; (2) patients who cannot complete an MRI examination because of fear; (3) be willing to abide by the contract. Exclusion criteria are as follows: (1) does not meet the diagnostic criteria for claustrophobia; (2) the patient is unwilling to cooperate with the practice of mindfulness technology to complete the examination; (3) the patient suffers from high-risk cardiovascular and cerebrovascular diseases. We divided the research subjects into two groups using a coin toss. The random allocation sequence was generated by YZ. Participants were enrolled by YC and SX, while SL and YL were responsible for assigning the participants to their respective interventions. There were 44 participants, comprising 21 men and 23 women. Their ages ranged from 22 to 65 years, with a mean age of 44.09 ± 11.5 years. In the control group, there were 42 participants, of whom 20 were men and 22 were women. The age range for this group was 19–68 years, with an average age of 46.40 ± 13.1 years. All subjects signed an informed consent form, and this study was approved by the medical ethics committee of our hospital.

### 2.2 Procedure

Adjustable lighting lamps, ventilation fans, alarm balloons, and air-conduction headsets were set in the scanning room. A player and a loudspeaker were provided in the operation and scanning frames, respectively. The control group adopted a conventional nursing method: filling out SAS and POMS-R before the intervention and then making patients fully aware of the importance of magnetic resonance examinations and cooperation, improving patients’ awareness of MRI examinations, and performing detailed nursing education. We used cotton balls to plug patients’ ears to reduce noise or soothing and relaxing music to relieve patients’ nervousness. Patients with severe symptoms may be accompanied by family members. The SAS and POMS-R were also completed after the intervention. On the basis of the nursing of the control group, the study group was given nursing intervention by mindfulness decompression therapy. The SAS and POMS-R were completed before the intervention, and then the mindfulness-based stress reduction method was practiced. Before practice, the researchers handed out audio and books and then informed the patients about the background, theoretical connotation, and practice process of mindfulness respiratory therapy. It can encourage patients to establish a calm and peaceful, go with the flow, optimistic, and confident mindfulness attitude. The specific measures are as follows: Patients feel relaxed and comfortable when sitting or lying down. Breathing was selected as the object of observation. Patients need to pay attention to every inhalation and exhalation and can focus on the nasal cavity, chest cavity, abdomen, or any sensitive part brought on by breathing in this process. Patients can gently put their hands on their abdomen and feel the ups and downs of their abdomen as they breathe. Patients also can memorize the “suck-breathe” in their minds. When they find their thoughts wandering, they return to the present and breathe again, without judgment or remorse. Patients have an independent space to practice by themselves. Patients complete the SAS and POMS-R again before they are examined, and then they enter the MRI room to complete the examination.

### 2.3 Observation indicators and evaluation criteria

Anxiety status: Zung’s self-rating anxiety scale (SAS) is used (23), and there are 20 questions in the scale. Criteria: the patient’s test score is less than 50 points, which means no anxiety; 50–60 points, mild anxiety; 60–70 points, moderate anxiety; and > 70 points, severe anxiety.

Emotional state: The profile of mood states revised (POMS-R) (24) is adopted, which is a highly reliable and valid emotional state rating scale. It includes six subscales (tension, depression, anger, energy, fatigue, and panic). Each subscale includes several adjectives (such as unhappy, unsatisfied, panicked, tired and sleepy, and listless). There are 65 adjectives in total. The words of each subscale are arranged in a mixed way, and each answer has five grades. The original scores of each subscale in five grades are accumulated separately, and the T score of each subscale is calculated by following the norm. The total score of emotional disorder (TMD) = the sum of the five negative emotional scores − the sum of the two positive emotional scores (energy and self-esteem) + 100. The higher the TMD score is the more negative the emotional state—that is, the more confused, annoyed, or unbalanced the mood state.

Examination completion effect: Marked effect: During the examination, patients can cooperate with the requirements of the medical staff to complete the examination in one attempt with a clear image. Effect: The examination process is not smooth, and it is necessary to communicate with the patient many times, but the patient can finally complete the examination with an effective image. Invalid: During the examination, the patient exhibits chest tightness, palpitation, and other clinical manifestations many times and cannot cooperate with the examination, and the image is invalid. Number of marked effects + effective number/total number * 100% = total effective rate of intervention.

Patients’ satisfaction with nurses’ psychological intervention technique: very satisfied, satisfied, generally satisfied, and dissatisfied. Very satisfied number + satisfied number/total number * 100% = satisfaction rate. High satisfaction means good intervention.

Comparison of the number of patients with severe autonomic nervous symptoms (such as dying feelings, pressure, dyspnea, sweating, syncope, and fear of beating the fuselage) during the examination: The fewer people with severe autonomic nervous symptoms, the more effective the intervention is.

### 2.4 Statistical analysis

Statistical analysis was performed using SPSS 18.0 software. The distribution of continuous variables was first assessed with the Shapiro–Wilk test. Depending on the distribution and type of group, either the dependent *t*-test or the Wilcoxon test was employed for the dependent groups, and the choice for independent groups was between the independent *t*-test and the Mann–Whitney test. Measurement data were represented as x ± s, while counting data were expressed in percentages (%). In each case, test statistics were provided, and an exact value of *p* was given. A significance level was set at a *p*-value of <0.05.

## 3 Results

There were 44 participants, comprising 21 men and 23 women. Their ages ranged from 22 to 65 years, with a mean age of 44.09 ± 11.5 years. In the control group, there were 42 participants, of whom 20 were men and 22 were women. The age range for this group was 19 to 68 years, with an average age of 46.40 ± 13.1 years.

**Figure 1:**
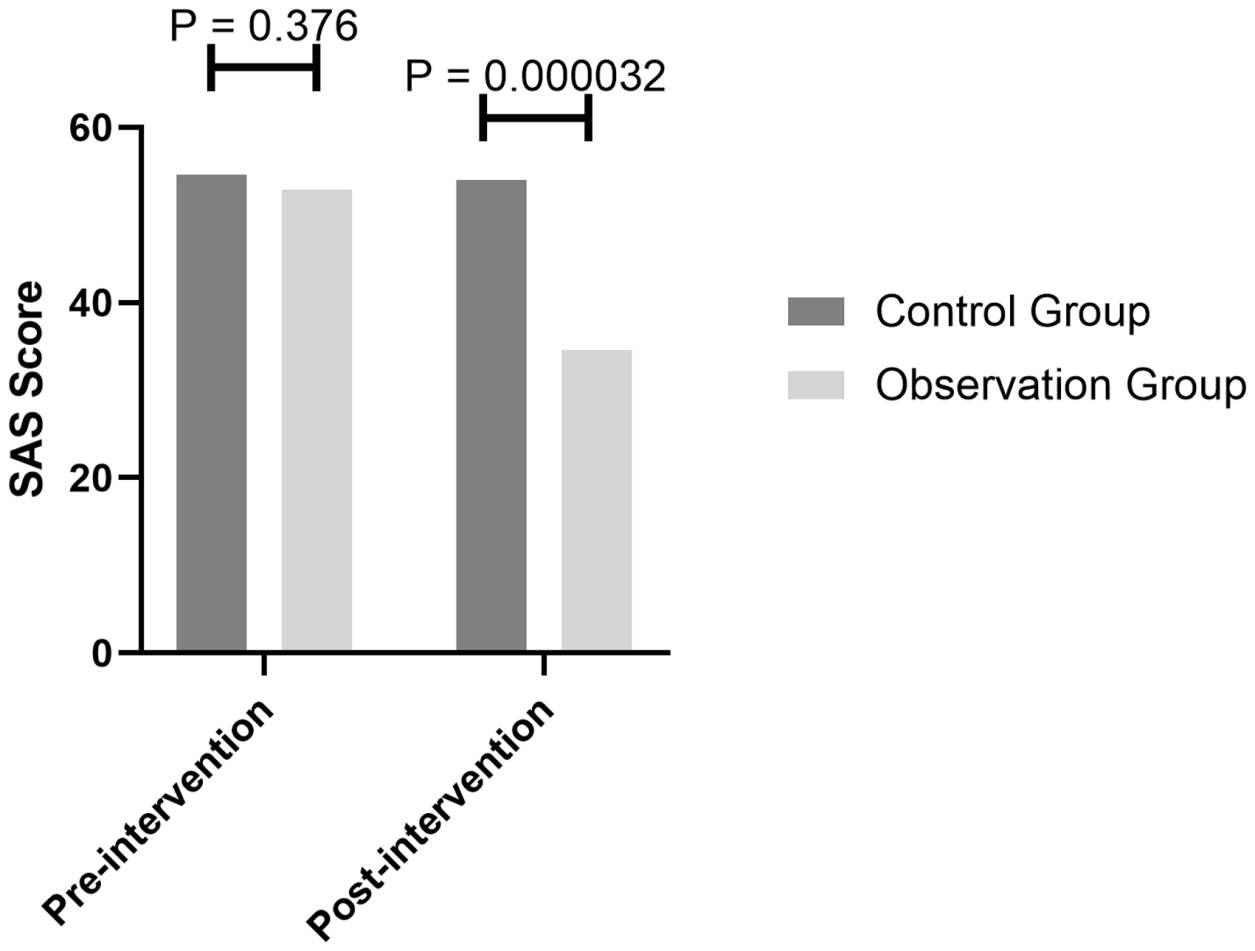
Comparison of the SAS scores between the two groups before and after the intervention.

After the mindfulness breathing intervention, the SAS score of the observation group decreased from a mild anxiety state (average 53) to a no-anxiety state (average 44), while the anxiety of the general psychological intervention group did not improve significantly and was still in a mild anxiety state (before 52/after 51). There was no significant difference in the anxiety state between the two groups before the intervention (*p* = 0.376), and there was a significant difference between the two groups after the intervention (*p* = 0.000032) (Figure 1).

**Figure 2:**
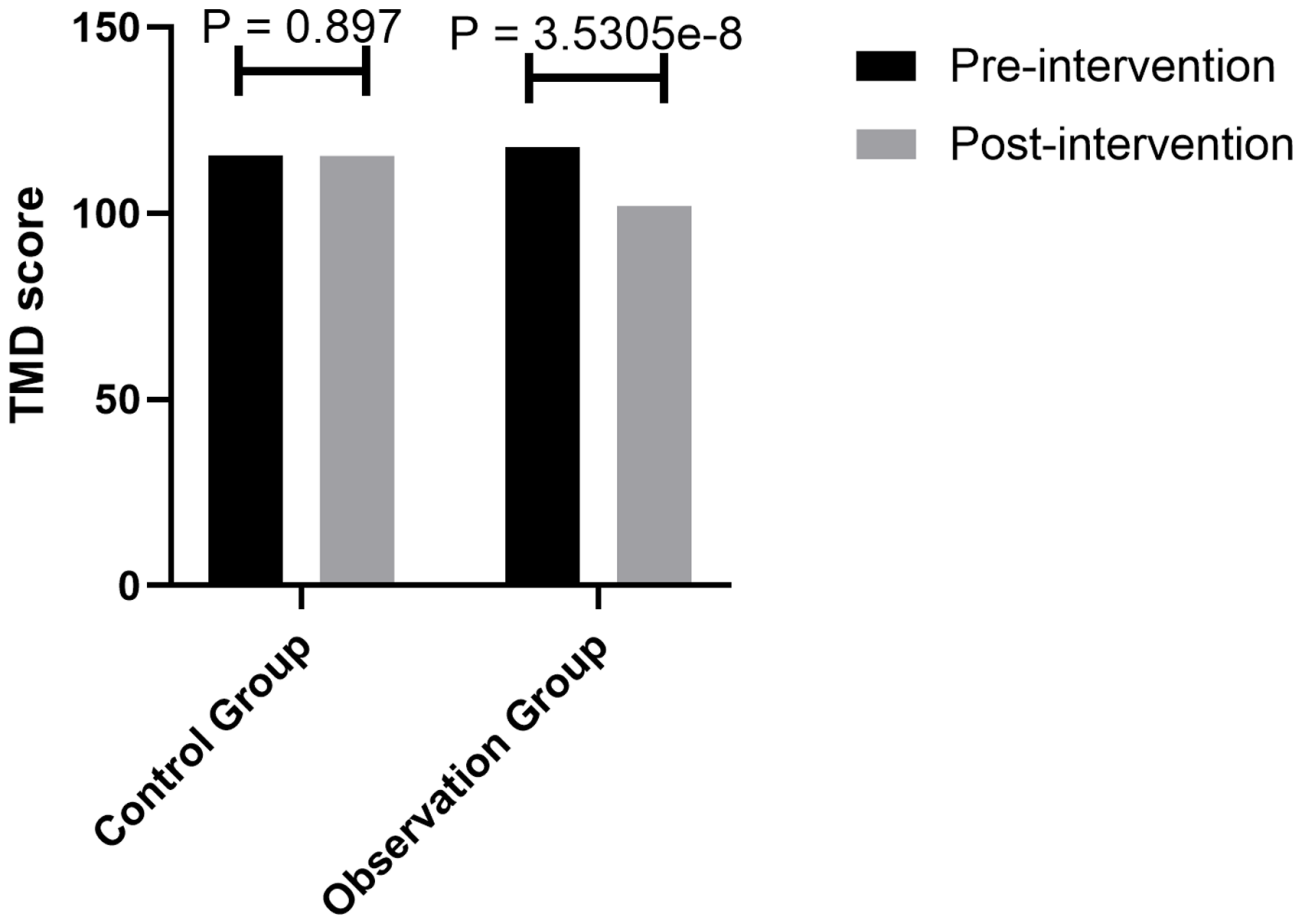
Comparison of the POMS-R scores between the two groups before and after the intervention.

The scores of the simplified POMS-R scale of the two groups showed that the total score of emotional disorder (TMD) and the score of negative emotion of the patients in the observation group decreased with the practice of mindfulness breathing, while the positive emotion increased due to the practice, and the difference was statistically significant (*p* = 3.5305e^−8^) (Figure 2).

**Figure 3:**
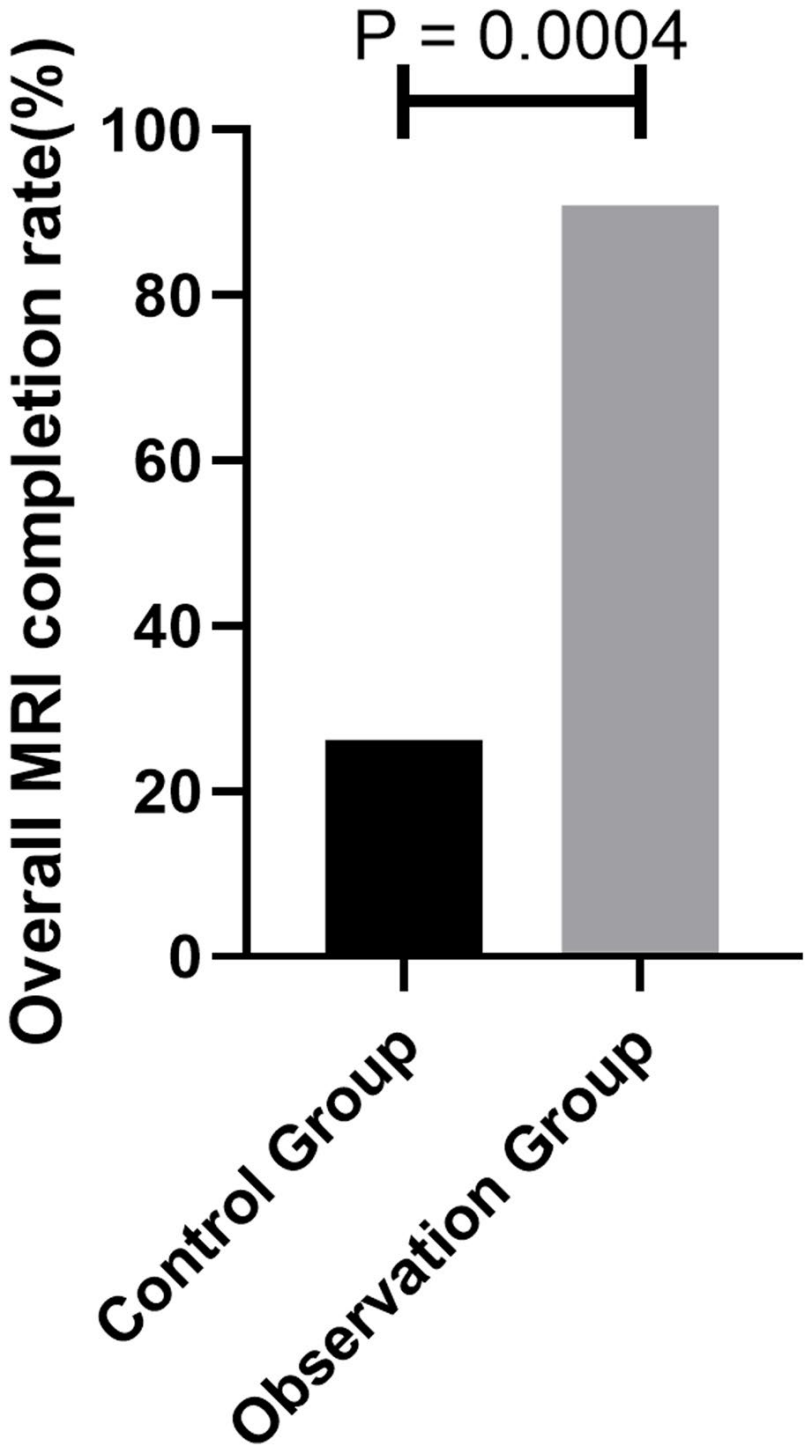
Comparison of the number of patients with MRI examination completion between the two groups.

After the mindfulness breathing intervention, the completion rate of magnetic resonance examination in the patients with claustrophobia in the observation group was 90.90%, which was statistically higher than 26.19% of the general psychological intervention (*χ*^2^ = 6.857, *p* = 0.0004) (Figure 3).

**Figure 4:**
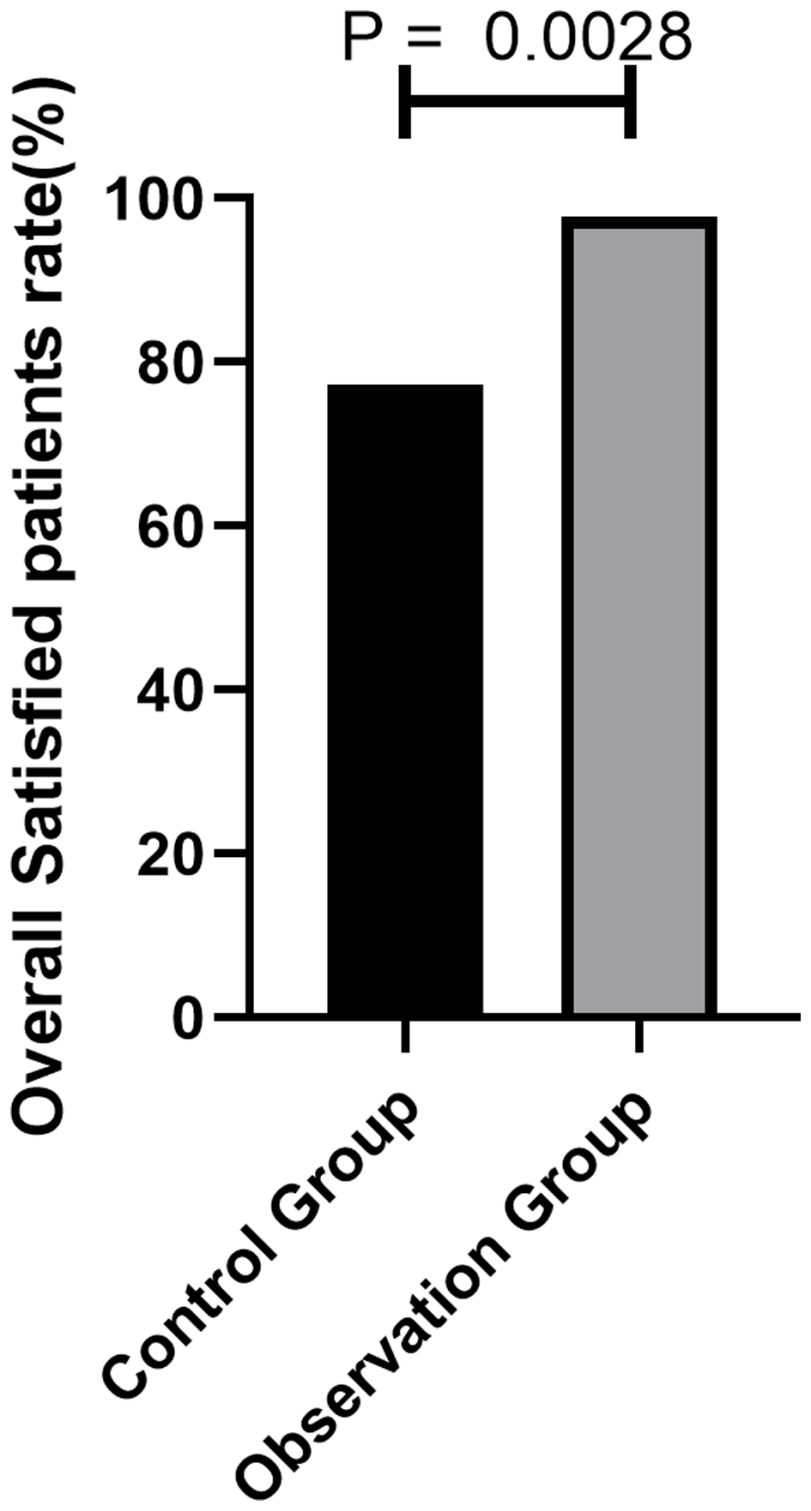
Comparison of the number of patients with MRI examination satisfaction between the two groups.

The satisfaction of the patients in the observation group with nurses’ psychological intervention technology was 97.72%, which was higher than 77.29% in the control group. The difference was statistically significant (*p* = 0.0028) (Figure 4).

**Figure 5:**
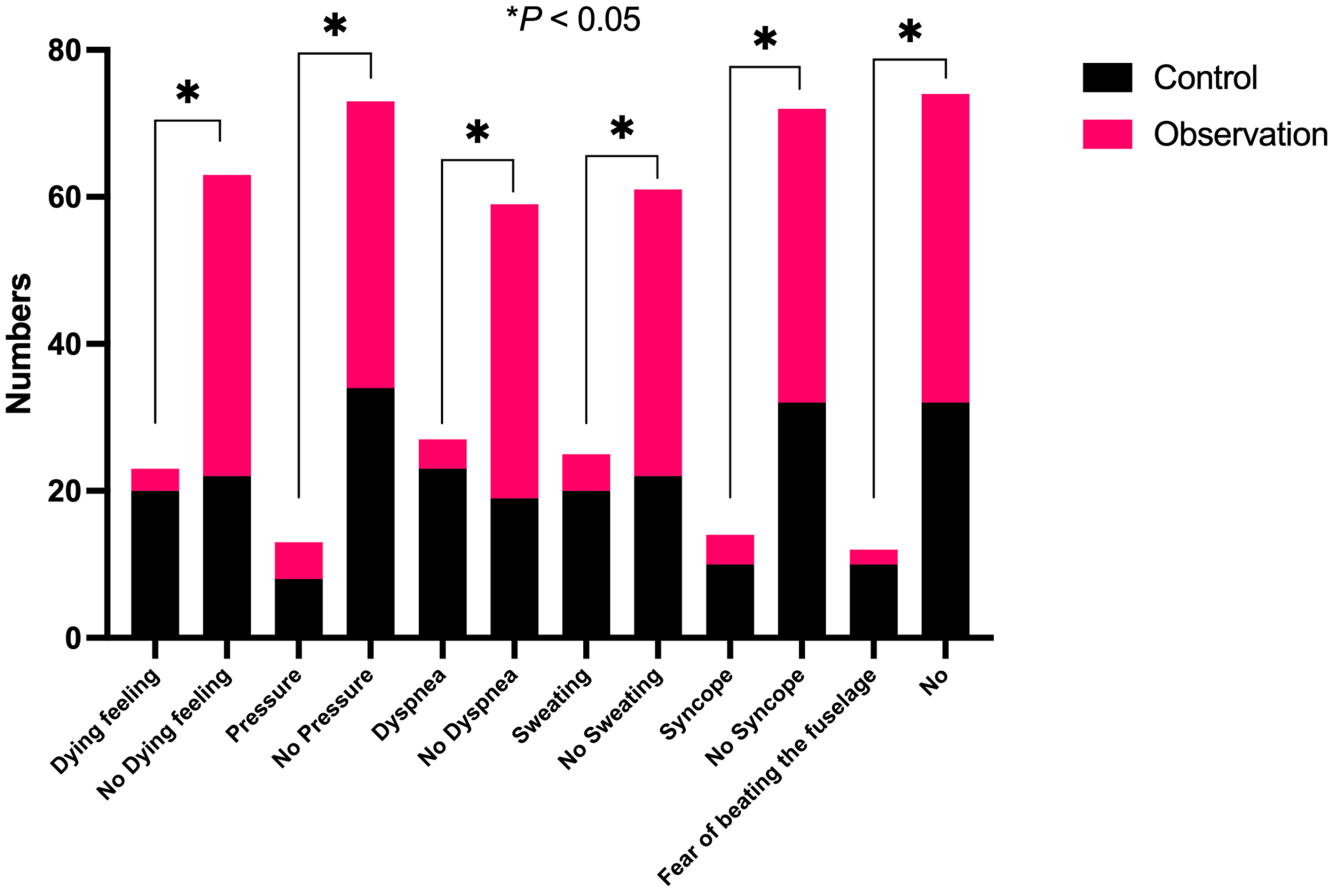
Comparison of the number of patients who developed severe autonomic nervous symptoms during the examination between the two groups.

The patients in the observation group had less severe autonomic nervous symptoms than those in the control group during the examination (*p* < 0.05) (Figure 5).

## 4 Discussion

Our study elucidated the potential benefits of mindfulness respiratory decompression therapy for claustrophobic patients undergoing MRI examinations. Within our cohort of 86 patients requiring MRI between January 2018 and December 2020, those who received mindfulness breathing techniques in addition to routine psychological care demonstrated a markedly enhanced intervention effect with a total effectiveness rate of 90.90%. This was considerably higher than the rate of the control group, which was 26.19%. Furthermore, severe autonomic nervous symptoms were significantly reduced in the observation group compared with the control group. Post-intervention assessments also revealed noteworthy reductions in both SAS and POMS-R scores in the observation group, underscoring the potential efficacy of the mindfulness-based approach. In essence, our findings suggest that mindfulness respiratory decompression therapy offers a promising avenue for enhancing the MRI experience of claustrophobic patients.

With the transformation of modern medical models into biological, psychological, and social medical models, increasing attention has been given to the influence of psychological factors on diseases (25). Mindfulness-based decompression therapy is an effective psychotherapy method that is safe, simple, and low in cost. It is neither subjected to technical limitations such as instruments nor restricted by the venue. Nurses have subjective operability (26).

In this study, mindfulness respiratory decompression therapy was used in the magnetic resonance examinations of claustrophobic patients. The results showed that the SAS scores of the patients in the observation group after the intervention by mindfulness respiratory decompression therapy were significantly lower than those in the control group. The anxiety of the patients in the observation group was reduced from mild anxiety to no anxiety, while the anxiety of the patients in the control group was still slight before and after the intervention. There was no obvious change. It is suggested that mindfulness respiratory decompression therapy can improve the anxiety of claustrophobic patients. This is similar to the research results by Reich et al. (27). Mindfulness decompression therapy can effectively improve pressure, anxiety, and depression in patients.

In addition to the assessment of anxiety, we also compared the various emotions of patients by evaluating the POMS-R. In this study, patients with claustrophobia were evaluated by POMS-R before and after the intervention. The results showed that there was no significant difference in the POMS-R scores between the two groups before the intervention, but the TMD value, tension, anger, fatigue, depression, panic, and other negative emotions in the observation group were significantly lower than those in the control group after the intervention. Meanwhile, positive emotions such as self-esteem and energy were significantly higher than those in the control group, and the difference was statistically significant. The TMD values of three claustrophobic patients before the intervention were 136.75, 128.59, and 130.78, respectively, and decreased to 88.63, 82.47, and 78.63, respectively, after the intervention. The TMD values of other patients decreased to different degrees. It is suggested that practicing mindfulness breathing and improving mindfulness levels can obviously improve the emotional state of claustrophobic patients, reduce negative emotions, and increase positive emotions.

The results showed that the completion rate of MRI examinations in the patients with claustrophobia in the observation group was 90.90% after the intervention, which was higher than that in the control group (26.19%). Patients’ satisfaction with nurses’ psychological intervention technique in the observation group was 97.72%, which was higher than that in the control group (77.29%). There were fewer patients with severe autonomic nervous symptoms in the observation group than in the control group. Mindfulness respiratory decompression therapy can reduce sympathetic nerve activity in claustrophobic patients, which is beneficial to the smooth completion of MRI examinations. Nurses pay attention to patients’ psychology accurately, which increases patients’ satisfaction. How does mindfulness respiratory decompression therapy accomplish this? Mindfulness emphasizes continuous attention and uncritical acceptance of internal and external stimuli in the moment. In this process, the individual’s perceptual sensitivity, attention, memory ability, emotional state, and emotional adjustment ability change significantly (20). The study confirmed that short-term mindfulness exercises can cause changes in brain information processing patterns (21). Regarding the influence mechanism of mindfulness, mindfulness mainly affects the lateral frontal lobe and basal ganglia. Some studies have found that mindfulness is related to the nerve remodeling of cortical-marginal circuits responsible for stress response and emotion regulation, and the decrease in gray matter volume in the hippocampus and caudate nucleus is related to idiosyncratic mindfulness. The stress-induced enhancement of the amygdala-anterior cingulate function can be reversed by mindfulness meditation practice. At the same time, for the implementation of mindfulness respiratory decompression therapy, we only need to prepare a relatively independent space to guide the patients to practice or watch the practice video and then let the patients practice by themselves. This method consumes a little time of staff and is easy for patients to accept, practice, master, and achieve good results.

This study had limitations. First, this was a single-center study, and the number of cases was relatively small. The indications, the adaptation population, and the maintenance time of the intervention through mindfulness respiratory decompression therapy need to be further studied. Considering the different stressors and stress levels faced by patients in different departments, research can be carried out for patients in different departments in future.

In conclusion, mindfulness respiratory decompression therapy is an economical, safe, effective, and comprehensive decompression method that plays the best role in nursing departments with medical imaging. It can improve patients’ satisfaction with nursing services, effectively help claustrophobic patients complete magnetic resonance examinations, improve patients’ satisfaction with nurses’ psychological intervention techniques, reduce the occurrence of severe autonomic nervous symptoms, reduce anxiety, and stabilize the mood state. At the same time, it can also provide timely and effective imaging diagnosis data for clinicians. Therefore, it is of great significance to both clinicians and the treatment of patients. After the intervention of mindfulness breathing technology, the number of patients with claustrophobia with severe autonomic nervous symptoms decreased significantly during magnetic resonance imaging.

## Data availability statement

The original contributions presented in the study are included in the article/supplementary material, further inquiries can be directed to the corresponding authors.

## Ethics statement

This study was approved by the Ethics Committee of Liuzhou People’s Hospital.

## Author contributions

YZ: Data curation, Formal analysis, Writing – review & editing. YC: Data curation, Writing – review & editing. SX: Data curation, Writing – review & editing. SL: Data curation, Writing – review & editing. YL: Data curation, Writing – review & editing. WZ: Formal analysis, Writing – original draft, Writing – review & editing. YX: Conceptualization, Writing – review & editing.
